# A Triumph for Pasteur

**Published:** 1887-09

**Authors:** 


### A TRIUMPH FOR PASTEUR.

The British Commission appointed last year to inquire into Pasteur's treatment of hydrophobia, have made their report to Parliament, from which we extract the following summary :

"It may hence be deemed certain that M. Pasteur has discovered a method of protection from rabies, comparable with that which vaccination affords against infection from small-pox. It would be difficult to overestimate the importance of the discovery, whether for its practical utility, or for its application in general pathology. It shows a new method of inoculation, or, as M. Pasteur sometimes calls it, of vaccination, the like of which it may become possible to employ for protection of both men and domestic animals, against others of the most intense kinds of virus. The duration of the immunity conferred by inoculation is not yet determined, but during the two years that have passed since it was first proved, there have been no indications of its being limited.

The committee think it, therefore, certain that the inoculations practised by M. Pasteur, have prevented the occurrence of hydrophobia, in a large proportion of those who, if they had not been so inoculated, would have died of that disease, and his discovery shows that it may become possible to arrest by inoculation, even after infection, other diseases besides hydrophobia. His researches have also added very largely to the knowledge of the pathology of hydrophobia, and supplied a sure means of determining, whether an animal which has died under suspicion of rabies, was really affected with that disease or not."

The *Medical News* says editorially :

The report of the British Hydrophobia Commission, constitutes the ablest defence of M. Pasteur's method, which has yet been made, and it is a cause for congratulation, that men so competent to observe facts and weigh evidence, have been able, after full investigation, to reach a unanimous conclusion as to the prophylactic value of the inoculations of Pasteur.

